# Auditory performance and cortical plasticity in blind individuals with sensorineural hearing loss: insights into dual sensory impairment

**DOI:** 10.3389/fnagi.2026.1769582

**Published:** 2026-10-01

**Authors:** Min Young Kwak, Jeong-Sug Kyong, Hyo Jung Choi, Yong-Hwi An, Hyun Joon Shim

**Affiliations:** 1 Department of Otorhinolaryngology-Head and Neck Surgery, Hallym University Dongtan Sacred Heart Hospital, Hwaseong, Republic of Korea; 2 Department of Radiology, Konkuk University Medical Center, Seoul, Republic of Korea; 3 Sensory Organ Research Center, Medical Research Institute, College of Medicine, Seoul National University, Seoul, Republic of Korea; 4 Department of Otorhinolaryngology-Head and Neck Surgery, Nowon Eulji Medical Center, Eulji University School of Medicine, Seoul, Republic of Korea; 5 Eulji Tinnitus and Hearing Research Institute, Nowon Eulji Medical Center, Seoul, Republic of Korea

**Keywords:** aging, blindness, evoked potential, hearing loss, neuroplasticity

## Abstract

**Background:**

Blind individuals with normal hearing frequently exhibit enhanced auditory abilities driven by compensatory cross-modal neuroplasticity. However, it remains unclear whether these compensatory advantages persist in individuals with dual sensory impairment. This study investigated auditory processing and cortical plasticity in early-blind, late-blind, and sighted individuals, all presenting with concurrent sensorineural hearing loss.

**Method:**

Participants completed auditory behavioral tests, including working memory, spectral ripple discrimination, temporal modulation detection, and sentence recognition in noise. Auditory oddball tasks assessed attentional and cognitive responses, and ERPs (N2, P3b) were recorded.

**Result:**

Results showed that late-blind individuals with hearing loss performed better than sighted individuals in digit span and temporal modulation tasks, but group differences in spectral resolution, speech recognition, and ERP components were largely not evident across conditions. Cortical source and functional connectivity analysis revealed distinct processing strategies, with sighted individuals recruiting frontal control regions under noise, whereas late-blind individuals relied on a temporal-parietal network.

**Conclusion:**

These findings indicate that in late-blind individuals, visual deprivation confers selective advantages over sighted peers in temporal processing and working memory even in the presence of age-related hearing loss, whereas broader perceptual benefits and electrophysiological enhancements are limited. Viewed in the context of prior literature in normal-hearing populations, these results suggest that compensatory cross-modal plasticity may be partially constrained when accompanied by concurrent sensorineural hearing loss.

## Background

Humans continuously perceive diverse information conveyed from the external environment via sensory convergence. The integration of multiple sensory inputs can enhance perceptual accuracy, improve behavioral outcomes, and lower discrimination thresholds for detecting stimuli (Stein et al., 2020). On the other hand, the human brain possesses a remarkable ability to adapt to sensory deficits through neuroplasticity, a process where neural circuits reorganize to compensate for the loss of a sensory modality (Pascual-Leone et al., 2005). In particular, the impact of visual or auditory deprivation on the remaining sensory performances has been extensively investigated.

Research has demonstrated that early-blind (EB) individuals who were either congenitally blind or lost their sight shortly after birth, with no visual experience retained in memory, exhibit a higher prevalence of absolute pitch (Hamilton et al., 2004) and superior abilities in various auditory tasks, such as pure-tone pitch discrimination (Voss and Zatorre, 2012), spectral ripple discrimination (Shim et al., 2019), and pitch discrimination (Wan et al., 2010) in both music and speech (Arnaud et al., 2018), as well as pitch-timbre categorization, compared to sighted individuals. These enhanced pitch discrimination abilities in blind individuals have been strongly associated with the extent of structural neuroplastic changes in the cortex (Voss and Zatorre, 2012). In addition, studies have shown a correlation between the age of onset or duration of blindness and pitch discrimination (Gougoux et al., 2004; Shim et al., 2019) and sound source discrimination (Voss et al., 2008). EB individuals also show improved temporal-order judgment (Weaver and Stevens, 2006), superior temporal auditory resolution through gap detection (Muchnik et al., 1991), enhanced detection of temporal modulation (Shim et al., 2019), and increased temporal attention in stimulus selection(Hötting and Röder, 2009). Unlike pitch discrimination, there was no evidence of a correlation between the duration of blindness and temporal resolution (Shim et al., 2019).

Although extensive research has documented the auditory and neural adaptations of EB individuals, studies focusing on LB populations remain relatively scarce. LB individuals who lost their vision in adulthood also demonstrate adaptive enhancements in auditory performance, particularly in spatial localization and temporal processing (Voss et al., 2004; Fieger et al., 2006; Voss et al., 2008). Regarding neural plasticity, whereas EB individuals primarily recruit the occipital cortex directly for auditory processing, LB individuals engage multimodal integration areas – such as the parietal-opercular region and the temporoparietal junction – along with residual neural networks (Ortiz-Terán et al., 2016). Importantly, neuroimaging literature indicates that the degree and pattern of this neural plasticity vary continuously as a function of the age of vision loss onset, even within the LB population (Collignon et al., 2013; Li et al., 2013). Specifically, while an earlier onset during late childhood promotes higher residual occipital cross-modal recruitment, a later onset shifts the compensatory mechanism toward an enhanced reliance on distributed parietal-temporal and cognitive-control networks (Ortiz-Terán et al., 2016). Studies indicate that, due to sensory compensation, both EB and LB individuals exhibit superior long-term memory and cognitive adaptability compared to sighted people, with EB individuals showing even greater advantages in pitch and sound source discrimination (Röder and Rösler, 2003; Voss et al., 2008). Other studies emphasize the importance of early visual experience in blindness. Late-blind individuals exhibit superior spatial cognitive abilities – such as proprioceptive reproduction and audio distance evaluation (Cappagli et al., 2017) – along with exceptional capacities in focusing auditory attention in the periphery (Fieger et al., 2006), identifying sound sources in horizontal space (Abel et al., 2002), and determining the relative distance of sound sources in far-auditory space (Voss et al., 2004).

Under normal-hearing conditions, previous studies from our laboratory have demonstrated that blind participants exhibit comparable or even superior performance relative to sighted participants under identical auditory-only conditions (Bae et al., 2022; Choi et al., 2024). Furthermore, when employing spectrally and temporally degraded vocoded speech, young EB participants demonstrated a clear advantage in speech recognition over sighted controls (Choi et al., 2024). However, that study examined young adults with normal hearing exposed to simulated vocoded speech, rather than elderly individuals with naturally occurring spectral or temporal resolution deficits. Blind individuals also experience hearing impairment as they age. However, it remains unclear whether their plasticity-driven auditory enhancements are preserved or diminished when age-related hearing loss occurs. Given their heavy reliance on hearing for external information, understanding how late-onset hearing loss affects these enhanced auditory abilities is critical for determining the durability and limits of neuroplasticity. In individuals with normal vision, age-related or acquired hearing loss induces a reduction of auditory cortical input, leading to cross-modal recruitment of the auditory cortex by the visual system alongside the compensatory activation of the frontal cortex (Glick and Sharma, 2017).

Dual sensory loss of vision and hearing has emerged as a critical health issue in aging societies (Heine and Browning, 2015). Therefore, we investigated the behavioral and neural profiles of auditory processing in elderly blind individuals presenting with age-related sensorineural hearing loss, focusing on neuroplastic dynamics under dual sensory impairment. Prior literature in normal-hearing blind adults demonstrates that visual deprivation induces adaptive neuroplasticity, fostering enhancements in temporal auditory processing and encouraging a top-down reliance on distributed parietal-temporal and cognitive-control networks. Grounded in this baseline framework, we hypothesized that such neuroplastic advantages would persist even in the presence of age-related sensorineural hearing loss, helping buffer against speech perception deficits under noisy environments. To systematically test this hypothesis, we aligned our test batteries across three distinct functional levels: peripheral psychoacoustic resolution, central cognitive-speech integration, and electrophysiological and network dynamics.

The selection of our behavioral test battery was motivated by our previous findings regarding the differential neuroplastic adaptation of spectral and temporal processing following visual deprivation (Shim et al., 2019). In normal-hearing individuals, visual loss leads to a selective behavioral advantage in temporal modulation detection (TMD) even in LB adults, whereas spectral ripple discrimination (SRD) enhancement appears restricted to EB individuals. This functional dissociation was further supported by correlation analyses in our prior study: SRD thresholds were significantly correlated with the duration of blindness, whereas TMD thresholds showed no significant correlation with the duration of blindness. To test whether this specific dissociation between spectral and temporal processing persists under actual sensory decline, we measured SRD and TMD in older blind individuals with sensorineural hearing loss. Furthermore, to determine how these basic psychoacoustic capacities interact with central cognitive resources during complex speech listening, we integrated measures of working memory, speech-in-noise perception.

At the electrophysiological and network level, we examined cortical auditory evoked potentials (CAEPs) to elucidate how higher-order cognitive processing is influenced by sensory degradation in blind individuals with hearing loss. Specifically, an auditory semantic oddball paradigm was employed to elicit the N2 and P3b components, which are known to reflect attentional control and conflict monitoring (N2) as well as stimulus evaluation and context updating (P3b). By integrating behavioral and electrophysiological measures, this study was a cross-sectional observational study investigating the effects of sensorineural hearing loss on auditory processing and cortical responses in blind and sighted elderly individuals.

## Materials and methods

### Participants

Three groups of participants with sensorineural hearing loss were recruited: individuals with early-blind (EB), late-blind (LB), and sighted individuals. All participants met the criteria for sensorineural hearing loss, defined as a bilateral, symmetrical, and progressive sensorineural hearing loss that primarily affects high frequencies, commonly associated with aging and without other identifiable pathological causes. The degree of hearing loss was greater than 40 dB but less than 70 dB at average hearing levels of 0.5, 1, 2, 3 kHz. Cases in which word recognition scores (WRS) were significantly lower than 70% were excluded from the study (Jerger and Jerger, 1988). WRS is defined as the percentage of monosyllabic words correctly identified and repeated when presented at 30–40 dB SL above the speech reception threshold.

The blind subjects, who were classified as 4 or 5 according to the 2006 World Health Organization (WHO) clinical diagnosis guidelines for visual impairment (category 4, with “light perception” but not “hand movement”; category 5, with “no light perception”), were included in this study (World Health Organization, 2006). The EB subjects were those born blind or became blind within days of birth, with no memory of vision. LB subjects who lost their vision after early childhood, typically after the age of 5 to 8 years, when basic visual development and visual-cognitive integration have already occurred. Only those presumed to retain memory of visual experience were included in the late-blind group. The LB participants in this study had experienced at least 10 years of visual deprivation. Table 1 shows the age of vision loss and cause of blindness in each blind participant.

**TABLE 1:** Demographic data on visual loss of blind individuals.

Subject	Age	Sex	Age of vision loss	Age of Hearing loss	Cause of blindness	Visual acuity
EB1	61	F	Birth	57	Unknown	No light perception
EB2	78	M	Birth	64	Unknown	No light perception
EB3	72	F	Birth	65	Unknown	No light perception
EB4	78	M	Birth	66	Unknown	No light perception
LB1	65	M	55	46	Optic atrophy	No light perception
LB2	74	M	30	72	Glaucoma	Light perception
LB3	62	M	57	48	Retinopathy	Light perception
LB4	64	M	15	57	Orbital trauma	No light perception
LB5	47	F	34	41	Glaucoma	No light perception
LB6	76	M	66	67	Cataract, glaucoma	No light perception
LB7	56	M	27	42	Retinal detachment, cataract	No light perception
LB8	49	M	11	45	Retinal detachment	Light perception
LB9	78	M	5	73	Unknown	Light perception
LB10	62	F	17	55	Retinopathy	No light perception
LB11	65	M	45	46	Retinopathy	No light perception
LB12	60	M	29	49	Optic nerve atrophy	No light perception
S1	62	M	N/A	47	N/A	Normal
S2	64	F		52		
S3	73	F		69		
S4	56	F		46		
S5	46	F		38		
S6	69	F		58		
S7	63	F		55		
S8	63	M		54		
S9	82	F		66		
S10	66	M		58		
S11	77	M		70		
S12	61	M		49		

EB, early-blind; LB, late-blind; M, male; F, female.

All subjects were right-handed, they had no significant otologic or neurological medical history, they were not hearing aid users. General cognitive status was assessed using the Mini-Mental State Examination (MMSE) as a screening measure. The MMSE for the blind (MMSE-blind) was derived by excluding eight vision-dependent items from the conventional MMSE, including object naming, following a written instruction, writing a sentence, copying a figure, and completing a three-step command (Reischies and Geiselmann, 1997; Busse et al., 2002). Consequently, the maximum possible score was 22 points, compared with 30 points for the standard MMSE. Based on previous literature, MMSE-blind scores > 17 were categorized as high, while scores ≤ 17 were considered low (suspected cognitive impairment). All participants enrolled in this study were in the high-scoring category.

The clinical characteristics of the participants in each group are presented in Table 2. Four EB (73.25 ± 8.02 years; M : F = 2 : 2) and twelve LB subjects (64.17 ± 9.70 years; M : F = 10 : 2) and twelve sighted subjects (65.17 ± 1.56 years; M:F = 5:7) participated in this study. There were no significant group differences in age (H(2) = 2.309, *p* = 0.315) or sex distribution (*p* = 0.100). Pure-tone averages (PTAs) were calculated separately for the right and left ears at 0.5, 1, 2, and 4 kHz (dB HL) revealing no significant group differences for either the right ear (H(2) = 2.502, *p* = 0.286) or the left ear (H(2) = 4.998, *p* = 0.082). A significant group difference was observed in the right-ear WRS (H(2) = 11.62, *p* = 0.003), while left-ear WRS did not differ significantly among groups (H(2) = 1.514, *p* = 0.469). *Post-hoc* pairwise comparisons (Bonferroni-adjusted significance level, *p* < 0.017) showed that the EB group had significantly higher right-ear WRS than the LB group (U = 4.000, Z = −2.520, *p* = 0.012) and the sighted group (U = 0.000, Z = −2.939, *p* = 0.003). The mean MMSE-blind scores of the three groups were 21.50 ±0.58 (EB), 22.00 ±0.00 (LB), and 21.33 ±1.56 (sighted). No significant difference in MMSE-blind scores was observed among the three groups (H(2) = 5.272, *p* = 0.072).

**TABLE 2:** Clinical characteristics for all subjects.

	Group			
Variables	EB	LB	Sighted	*p*-value
Age	73.25 ±8.02	64.17 ±9.70	65.17 ±1.56	0.322
Sex (M : F)	2 : 2	10 : 2	5 : 7	0.419
PTA				
Right	49.69 ± 6.24	55.73 ± 12.28	53.98 ± 4.96	0.438
Left	45.00 ± 20.54	59.69 ± 17.82	47.27 ± 7.21	0.087
WRS				
Right	100.00 ±0.0	90.33 ±10.12	81.5 ±8.89	0.003*
Left	91.00 ±10.51	83.50 ±8.89	78.66 ±10.35	0.469
Blind duration	72.20 ±7.33	30.58 ±20.24	−	−
HL duration	10.25 ±4.57	10.75 ±5.67	11.00 ±3.41	0.962
MMSE	21.50 ± 0.58	22.00 ± 0.00	21.33 ± 1.56	0.072

EB, early-blind; LB, late-blind; M, male; F, female; PTA, pure tone audiometry; WRS*, word recognition score; HL, hearing loss; MMSE, Mini-Mental State Examination.

This study was conducted in accordance with the Declaration of Helsinki and was approved by the Institutional Review Board of Eulji Medical Center. Written informed consent was obtained from all participants. For blind participants, the contents of the consent form were explained verbally in the presence of an impartial witness, after which the participants provided written consent. A copy of the signed form was given to each participant.

### Auditory behavioral tests

The EB, LB, and sighted subjects performed five behavioral tests: digit span test (Wechsler, 1987), temporal modulation detection (TMD) (Won et al., 2011), spectral ripple discrimination (SRD), Korean matrix sentence recognition in noise (Kim and Lee, 2018; Jung et al., 2022), monosyllable recognition in noise (Shim et al., 2019). The sighted participants performed the task while fixing their gaze on a central cross. All tests were performed in a soundproof room using an audiometer (Madsen Astera 2; GN Otometrics, Taastrup, Denmark) with the subjects wearing insert earphones (Etymotic, ER-3A). All auditory stimuli were calibrated and presented at a fixed level of 70 dB HL. Prior to test administration, an individualized audibility check was routinely performed with test tokens to verify that the stimuli were clearly audible and comfortably heard by all participants.

#### Digit span test

A digit span task was employed to assess the role of working memory in central auditory processing. The stimuli consisted of sequences of digits ranging from 1 to 9, with the number of digits increasing stepwise from three to ten. Each sequence length was presented twice, and the sequences were generated randomly. The span threshold was defined as the maximum sequence length at which the participant made fewer than two errors. Stimuli were presented at 70 dB HL, with a 1-s interval between sequences. Participants were asked to repeat the digits in both forward (SPAN-F) and backward order (SPAN-B).

#### Temporal modulation detection (TMD) test

For each trial, sinusoidal amplitude modulation was applied to a newly generated wideband white noise carrier. The modulation frequency was set at 100 Hz, and all stimuli were presented at an intensity of 70 dB HL. Both modulated and unmodulated (steady) signals had a duration of 1 s and were shaped using 10-ms linear onset and offset ramps. These two types of stimuli were presented sequentially without a silent gap between them.

TMD thresholds were obtained using a two-interval, two-alternative forced-choice (2-AFC) paradigm. In each trial, one interval contained modulated noise, while the other contained steady noise. Participants were instructed to identify the interval (first or second) that they perceived as modulated by pressing the corresponding number on the keyboard (e.g., pressing “2” if the second interval contained the modulated signal).

A two-down, one-up adaptive tracking procedure was used to estimate the TMD threshold. Thresholds were expressed in decibels relative to 100% modulation, calculated as 20log_10_(mi), where mi denotes the modulation index. The tracking began at full (100%) modulation depth, with a step size of 4 dB for the first four reversals and 2 dB for the subsequent ten. For each run, the threshold was defined as the average modulation depth across the last ten reversals. Final TMD thresholds were determined by averaging the results of three independent runs.

#### Spectral ripple discrimination (SRD) test

The SRD test was performed as described by Won et al. (2007). A ripple depth of 30 dB was employed for the stimuli. In the reference signal, the initial phase of the full-wave-rectified sinusoidal spectral envelope was set to 0 radians, whereas the oddball stimulus began at a phase of π/2 radians. Thus, the perceptual contrast between the two stimuli was created by differing starting phases (Supplementary Figure 1). The constituent pure tones were spaced logarithmically across a frequency range of 100 to 4991 Hz, which ensured distinct spectral peaks and troughs, especially at higher ripple densities. The spacing of ripple peaks also followed a logarithmic frequency scale. The stimuli had a total duration of 500 ms and were ramped with 150 ms linear rise/fall times. The stimuli were processed using a speech-shaped long-term average filter generated with CoolEdit 2000 (Halling, 2000), with parameters specified in accordance with the findings of Byrne et al. (1994). Stimuli were presented at a fixed level of 70 dB HL. SRD thresholds were obtained using a three-interval, three-alternative forced-choice (3-AFC) paradigm with an adaptive two-up, one-down procedure. Stimulus order was randomized, and participants identified the “oddball” stimulus – i.e., the one that differed from the other two – by pressing the corresponding button on a custom response device. For instance, if the oddball was in the second interval, participants pressed “2” on the keyboard.

Ripple density was adaptively adjusted from 0.125 to 11.314 ripples per octave in equal-ratio steps of 1.414. Each adaptive run included 13 reversals targeting the 70.7% correct response rate. In each 3-AFC trial, signal levels were randomly attenuated by 1–8 dB in 1 dB steps. SRD thresholds were defined as the mean ripple density of the final 8 reversals. Higher thresholds reflected better spectral resolution. The final value was obtained by averaging two or three runs per participant.

#### Sentence recognition in noise

Sentence-in-noise recognition was assessed using the Korean Matrix sentence recognition test (Luck, 2014). The Korean Matrix sentences employed in the test were designed to be semantically unpredictable while maintaining a consistent syntactic format, comprising five components: a name, adjective, noun, numeral, and verb. Each sentence was constructed from a fixed matrix of 50 base words, with 10 options available in each lexical category. The design rationale and practical implementation of Matrix sentence-in-noise recognition tests have been thoroughly outlined in prior literature (Tadel et al., 2011). The Korean Matrix sentence-in-noise test was conducted using two forms of background noise: speech-shaped noise (SSN) and the International Speech Test Signal (ISTS). The SSN was created by overlapping multiple Korean Matrix sentences, resulting in a long-term spectral profile that closely matched that of the speech stimuli. The ISTS noise (Holube et al., 2010) is considered non-intelligible speech noise because it consists of randomly remixed speech segments (100–600 ms) from six languages, which are spoken by six different female talkers reading “The North Wind and the Sun”. The Korean Matrix sentence recognition task was administered using the Oldenburg Measurement Applications (OMA) software developed by HörTech GmbH (Oldenburg, Germany). Test sentences and noise were delivered via a Fireface UCX converter and played through a loudspeaker positioned 1 meter in front of the subject. Noise was fixed at 65 dB HL, and sentence levels were adaptively adjusted using the maximum likelihood estimator (Brand and Kollmeier, 2002). Consequently, we measured the speech recognition thresholds of 50% intelligibility by measuring the SNRs required to achieve 50% recognition.

#### EEG recording

Neural activity was recorded using 31 sintered Ag-AgCl electrodes positioned according to the international 10–20 system (Klem, 1999), with reference at FCz, mounted in a 32-channel elastic EEG cap. Data acquisition was performed using the actiCHamp recording system and BrainVision Recorder Professional software (Version 1.23.0001; Brain Products GmbH, Munich, Germany) in a dimly lit, sound-attenuated, and electrically shielded room. Electrooculogram (EOG) and electrocardiogram (ECG) channels were included to monitor eye movements and cardiac activity. EEG signals were digitized in real-time at a 1000 Hz sampling rate. All channels were re-referenced offline to the common average to avoid spatial bias. The ground electrode was positioned between Fp1 and Fp2. Online filtering included a 0.5 Hz high-pass and a 70 Hz low-pass filter, along with a 60 Hz notch filter to eliminate powerline interference. Scalp electrode impedance was kept below 5 kΩ throughout the recording, as per the manufacturer’s guidelines. In the semantic oddball task, participants were presented with either animal-related words or semantically plausible but non-animal words. Animal stimuli (e.g., mouse, snake, bear) accounted for 70% of all trials, and all target words were monosyllabic in Korean. The remaining 30% of the stimuli consisted of monosyllabic non-animal words that were phonetically similar to animal words but did not belong to the animacy category. Participants were seated comfortably in a sound-attenuated booth and listened to randomized presentations of both animal and non-animal words. Prior to the task, participants were informed that animal words would appear throughout the session and instructed to respond quickly and accurately only when a non-animal word was presented. This design encouraged sustained attention, as participants had to monitor the stream of stimuli and respond by pressing a button when a non-animal word was detected.

For each SNR condition (−6, 0, +6 dB, and clear speech), 210 animal words and 90 non-animal words were presented across 10 blocks, totaling 1,200 trials per participant. Stimuli were delivered with a fixed inter-stimulus interval of 2000 ms, incorporating a jitter of 2–5 ms to reduce predictability. The order of stimulus presentation was randomized within each block, and the sequence of blocks was counterbalanced across participants using E-Prime software (version 3, Psychology Software Tools, Sharpsburg, PA). Participants were given a 3-min break after each block. Prior to the EEG recording session, a familiarization phase was conducted to ensure task comprehension and minimize muscular tension. All auditory stimuli were presented at a constant intensity of 70 dB HL via insert earphones. Sighted participants were instructed to fixate on a central cross (+) and minimize eye movements, while blind participants were similarly encouraged to maintain a steady gaze. Eye-movement artifacts were removed using independent component analysis (ICA) during preprocessing. All behavioral tests were conducted under audio-only conditions to ensure a consistent experimental environment across groups.

#### Cortical representations of higher level of speech processing

The N2 and P3b components observed in electroencephalographic (EEG) recordings are indicative of higher-order cognitive functions such as novelty detection, conflict monitoring, stimulus identification, attentional allocation, inhibition control, and the regulation of task-relevant processing. These components are typically evoked using an oddball paradigm, in which rare deviant targets are interspersed among frequent standard stimuli (Kujala et al., 1995). The N2 component, typically emerging approximately 200 ms post-stimulus, reflects neural activity associated with lexical access and semantic categorization processes (Finke et al., 2016). The P3b component, occurring around 300 ms after stimulus onset, is linked to working memory updating; delays in its latency may suggest slower stimulus evaluation or cognitive processing speed (Henkin et al., 2015).

#### Data processing

EEG data were preprocessed and analyzed using Brain Vision Analyzer (version 2.0, Brain Products) and the EEGLAB v2021 toolbox (Delorme and Makeig, 2004) running in MATLAB environments (versions 2014a and 2019b). A 0.1 Hz high-pass Butterworth filter (12 dB/oct roll-off) and a 50 Hz low-pass Butterworth filter (24 dB/oct roll-off) were applied to the continuous EEG signals. The first three trials from each condition per participant were excluded prior to analysis. The data were subsequently downsampled to 256 Hz (Grandchamp and Delorme, 2011).

Independent component analysis (ICA) was used to identify and remove artifacts related to eye blinks and body motion, typically eliminating four components on average (range: 3–6). The components removed mostly included 2 eye movements (vertical/horizontal movements or eye blinks etc.). Rest of the movements were head- or body-related movements in most of the participants/conditions. The data were then re-referenced to the common average (Makeig et al., 1997). Epochs were extracted from −200 ms to 1,000 ms relative to stimulus onset, and baseline correction was applied using the pre-stimulus interval.

Before averaging, any channels exhibiting poor signal quality were interpolated via a spherical spline interpolation method (Perrin et al., 1989). Epochs exceeding ± 70 μV at any electrode were excluded. This data cleaning process also included the rejection of trials in which button-pressing occurred during stimulus presentation or exceeded 2000 ms after stimulus onset, as the interstimulus interval was set at 2000 ms. Finally, 69.98 ± 8.42 % of trials for EB, 61.36 ± 2.39 % for the sighted group, and 72.10 ± 6.87 % for LB survived artifact rejection and were valid for further analysis. An average wave file was generated for each subject for each condition. Latency ranges for the N2 and P3b components were established based on the grand average waveform across all conditions and participants, following prior literature (Finke et al., 2016). The N2 was defined as occurring between 300 and 805 ms post-stimulus, and P3b between 315 and 725 ms, as illustrated in Figure 1. Difference waveforms were derived by subtracting responses to target stimuli from those to standard stimuli within each condition (Deacon et al., 1991). Instead of measuring the time of a single maximal peak, which is highly susceptible to high-frequency noise and latency variability, we used half-area fractional area latency calculation identifying the exact time point where the integrated area under the curve reaches exactly half of the total area of a component of interest. This method is known to equalize variance and provide a more stable and robust temporal measure than peak latency (Luck, 2014; Finke et al., 2016). Specifically, the latency was defined as the time point at which 50% of the area under the curve had accumulated. N2 amplitudes were measured by averaging signals from frontocentral electrodes (Fz, FC1, FC2, and Cz), and P3b from parietal electrodes (CP1, CP2, P3, P4, and Pz), as outlined by Finke et al. (2016).

**FIGURE 1:**
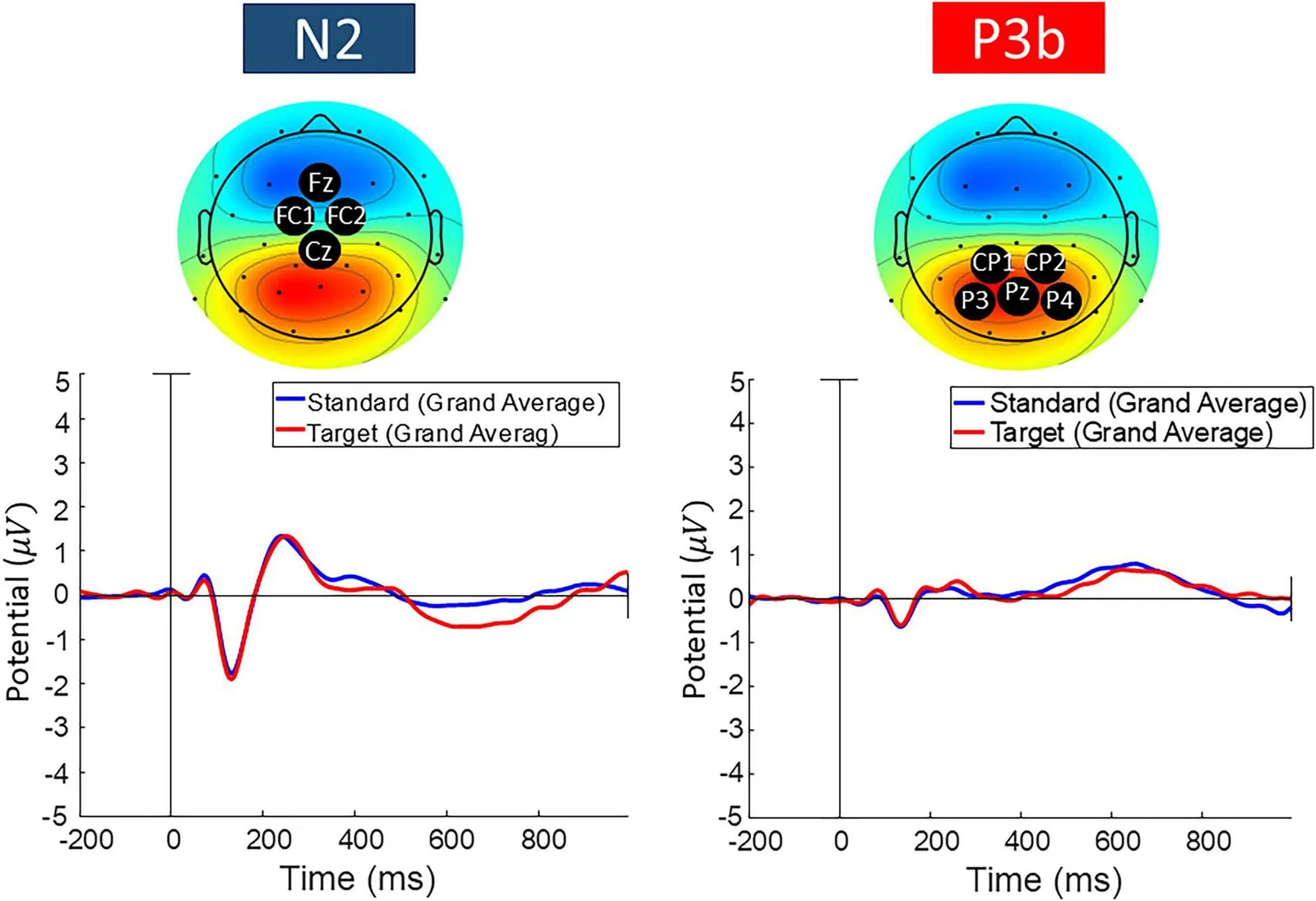
N2 and P3b Analysis. N2 was measured by averaging waveforms of four frontocentral electrodes (Fz, FC1, FC2, and Cz). P3b was measured by averaging waveforms of five parietocentral electrodes (CP1, CP2, P3, P4, and Pz).

To localize cortical sources associated with significant scalp-level effects, source reconstruction was performed using Brainstorm (Tadel et al., 2011) and exact low-resolution brain electromagnetic tomography (sLORETA) software (Pascual-Marqui, 2002), within the time windows showing statistical significance. We allocated 5,000 permutations for the log F-ratio test to achieve high statistical precision (down to *p* = 0.0002) for stringent thresholds like Bonferroni correction, and 1,000 permutations for the non-parametric surrogate test to balance computational efficiency for standard significance levels (*p* = 0.001), following established guidelines (Pascual-Marqui, 2002). Functional connectivity among 29 predefined brain nodes was further assessed using the Brain Connectivity Toolbox (BCT) (Rubinov and Sporns, 2010), and the results were visualized with BrainNet Viewer^1^. Binary undirected network measures were used with an epoch duration of 2000 ms in the −6 dB SNR condition. A symmetric orthogonalization function was applied to mitigate spurious connectivity. Degree values across predefined nodes were extracted, and connections with *p* < 0.05 were visualized in Figure 7.

### Statistical analysis

Group differences in age, PTA, WRS, MMSE-blind scores, behavioral measures, and N2/P3b amplitudes and latencies were assessed using Kruskal-Wallis tests. Sex distribution across the three groups was evaluated using the Fisher-Freeman-Halton exact test due to small expected cell counts. *Post-hoc* pairwise comparisons were conducted using Mann–Whitney U tests with Bonferroni correction. For the three pairwise group comparisons, the adjusted significance level was set at *p* < 0.017.

Pearson correlation analyses between behavioral performance and ERP measures across the four SNR conditions were also corrected for multiple comparisons using the Bonferroni method. For source analysis, voxel-by-voxel group comparisons using the log F-ratio were evaluated using a non-parametric test with 5,000 permutations, as described in previous literature (Volpe et al., 2007). Functional connectivity between the LB and sighted groups under the −6 dB SNR and clear-speech conditions was evaluated for exploratory purposes using a non-parametric surrogate test with 1,000 permutations, and the findings were interpreted descriptively rather than inferentially. All other statistical analyses were conducted using IBM SPSS Statistics (version 27.0; IBM Corp., Armonk, NY, USA).

## Result

### Behavioral psychoacoustic tests

Figure 2 presents the results of behavioral psychoacoustic assessments. Given that the right-ear WRS significantly differed among the groups, we conducted additional statistical analyses for all behavioral auditory tasks by controlling for WRS as a covariate. The Kruskal–Wallis test on these residuals revealed that group differences in the SPAN-F remained significant even after accounting for right-ear WRS (H(2) = 12.416, *p* = 0.002). *Post-hoc* pairwise comparisons were conducted using the Mann-Whitney U test with Bonferroni correction to control for multiple comparisons. With the adjusted significance threshold set at *p* < 0.017, significant differences were observed between the LB and sighted groups in the SPAN-F, with the LB group showing significantly higher scores than the sighted group (U = 10.000, Z = −3.583, *p* < 0.001). In the TMD, a significant difference among the three groups remained (H(2) = 9.966, *p* = 0.007), and *post-hoc* analysis using the Mann-Whitney U test with Bonferroni correction revealed that the LB group outperformed the sighted group (U = 27.000, *p* = 0.009), indicating superior temporal auditory resolution in the late-blind participants. In contrast, the SRD showed no significant differences among the groups (H (2) = 3.773, *p* = 0.152), and the sentence-in-noise test indicated no group differences in recognition performance for either SSN (H (2) = 0.434, *p* = 0.805) or ISTS (H (2) = 0.313, *p* = 0.855).

**FIGURE 2:**
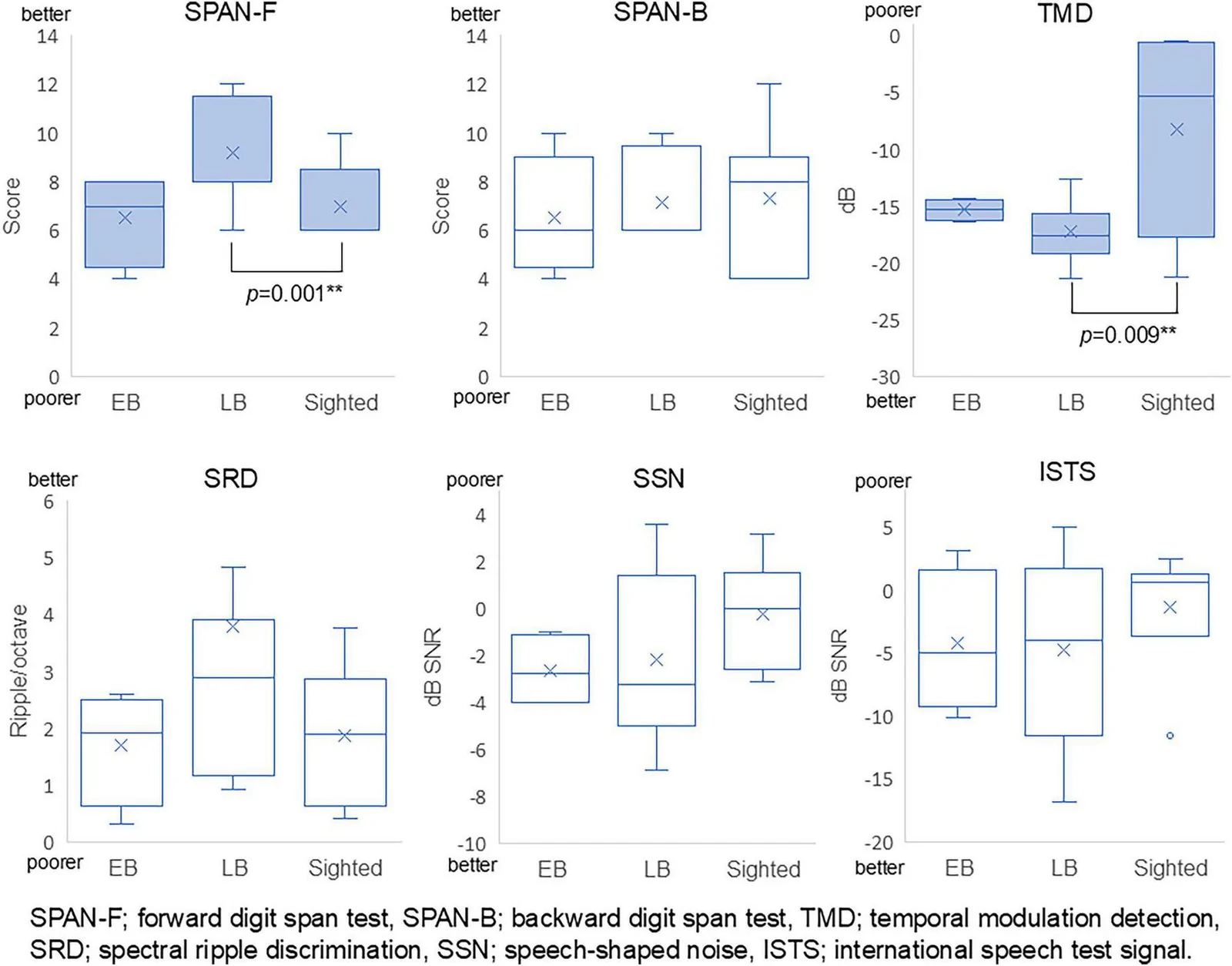
Comparison of mean data from the psychoacoustic tests (SPAN, TMD, SRD, sentence recognition in noise) between the blind individuals (early-blind; EB, late-blind; LB) and sighted individuals. SPAN-F, forward digit span test; SPAN-B, backward digit span test; TMD, temporal modulation detection; SRD, spectral ripple discrimination; SSN, speech-shaped noise; ISTS, international speech test signal.

#### Accuracy and ERP (N2, P3b) amplitude and latency in the oddball task

Figure 3 shows comparisons of area peak latencies and amplitudes for the N2 and P3b difference waveforms across groups and conditions. Figure 4a shows the group accuracy in the oddball task across SNR conditions, with no significant group differences found under any condition. In the measurement of N2 and P3b components, we observed a pattern in which the amplitude decreased, and latency increased as the SNR condition became more challenging. A Friedman test was conducted to examine differences in N2 peak latency (χ^2^(3) = 51.95, *p* < 0.001), N2 amplitude (χ^2^(3) = 50.15, *p* < 0.001), P3b latency (χ^2^(3) = 39.00, *p* < 0.001), and P3b amplitude (χ^2^(3) = 60.97, *p* < 0.001) across four SNR conditions (−6 dB, 0 dB, +6 dB, and clear sound). To account for differences in right-ear WRS, group comparisons were performed using residuals obtained by regressing out WRS from the dependent variables. A Kruskal–Wallis’s test showed a significant difference in N2 amplitude only in the –6 dB SNR condition among the three groups (H(2) = 8.211, *p* = 0.016), and *post-hoc* analysis revealed a lower amplitude in the LB group compared to the sighted group (U = 25.000, Z = –2.525, *p* = 0.011, Figure 4b), which remained statistically significant after Bonferroni correction (adjusted significance threshold: *p* < 0.017). No significant differences in N2 latency, P3b latency and P3b amplitude were observed among the three groups across SNR conditions (*p* > 0.05 Figure 4c).

**FIGURE 3:**
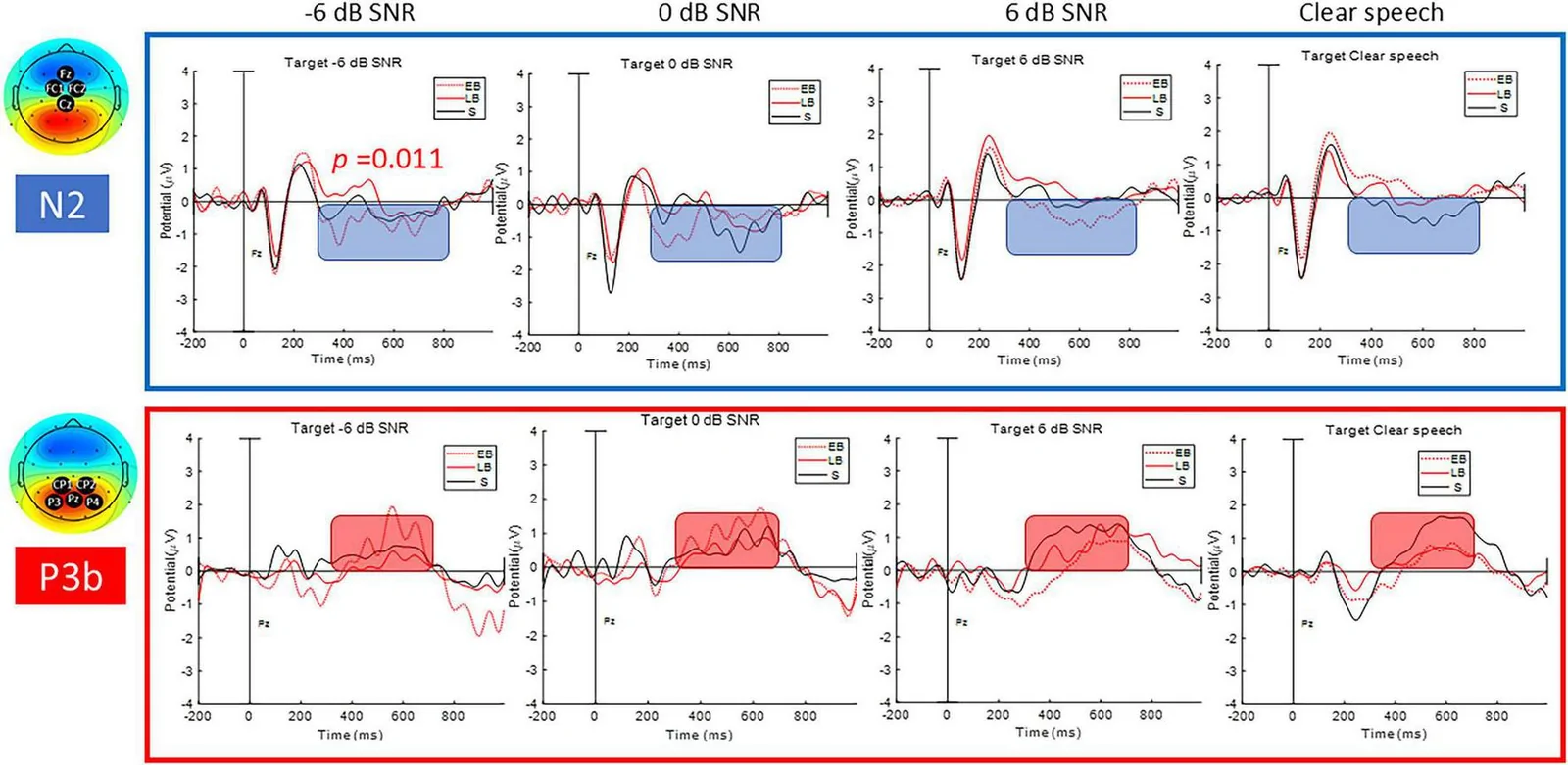
Electrophysiological N2 and P3b responses elicited by the oddball paradigm. Grand average waveforms of target in conditions of −6, 0, +6 dB SNR and clear speech across groups (EB: early blind, LB: late blind, (S) sighted) in N2 (upper panel, 300∼805 ms post onset of a stimulus) and P3b (lower panel, 315∼725 ms post onset of a stimulus). The time windows for analysis were defined based on the grand average waveform in all conditions across groups. The shaded blue square is the time window for N2, and the shaded red square is the time window for P3b. Waveforms were from the electrodes Fz (N2) and Pz (P3b) for the ease of illustration.

**FIGURE 4:**
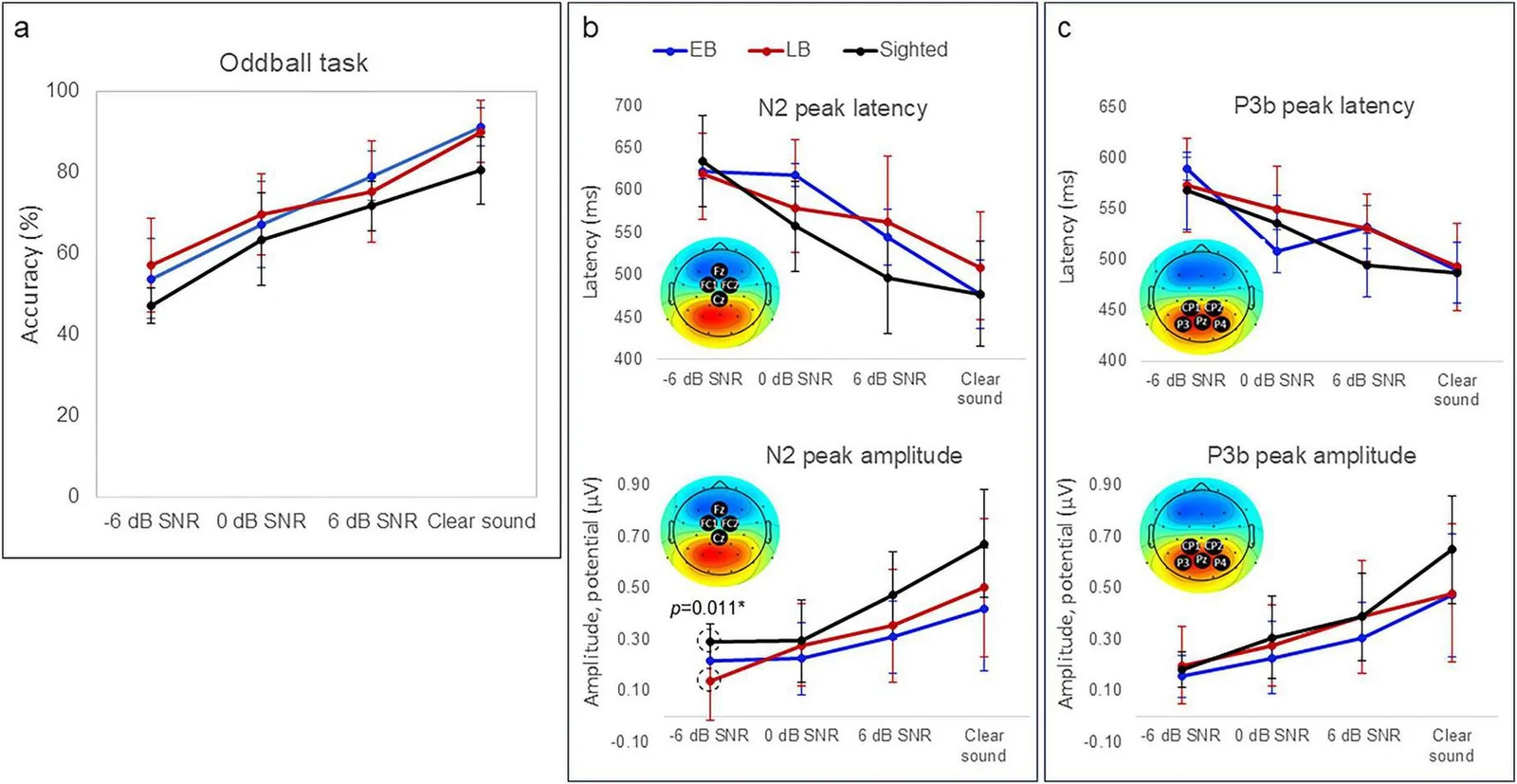
Oddball task accuracy and ERP measures. **(a)** Average accuracy (%) for the oddball task in three groups. **(b, c)** Amplitude and latency comparisons of N2 and P3b between the blind individuals (early-blind; EB, late-blind; LB) and sighted individuals.

#### Correlation of neural response with behavioral data

We examined the correlations between oddball task accuracy and neural responses (N2 and P3b), in terms of latency and amplitude, across different SNR conditions. Among the 16 comparisons, only the correlation between P3b latency and accuracy under the 0 dB SNR condition remained statistically significant after Bonferroni correction (*p* < 0.003*). The strength and direction of the correlations varied depending on SNR and ERP component, with some trends observed in the 0 and 6 dB conditions. Detailed Pearson’s *r* and corresponding *p*-values are shown in Table 3.

**TABLE 3:** Correlations between oddball task accuracy and ERP components (N2, P3b) under different SNR conditions.

SNR Conditions		N2 peak		P3b peak	
		Latency	Amplitude	Latency	Amplitude
−6 dB SNR	R	−0.238	0.249	−0.095	−0.251
	*p*	0.231	0.211	0.638	0.207
0 dB SNR	R	0.449	−0.128	**0.545**	0.012
	*P*	0.017	0.516	0.003*	0.953
6 dB SNR	R	0.460	−0.21	0.222	−0.445
	*P*	0.014	0.284	0.256	0.018
Clear	R	0.154	0.057	0.17	0.048
	*P*	0.461	0.788	0.416	0.819

Pearson’s correlation coefficients (r) and their associated *p*-values are presented. Bold values indicate statistically significant correlations. Given the number of SNR conditions (*n* = 4), Bonferroni correction was applied to control for multiple testing, setting the adjusted significance threshold at *p* < 0.0125 Source localization and network connectivity.

The significant stronger sources in the sighted group resulted in the frontal region in the left hemisphere when compared with the LB group in the –6 dB SNR and clear speech conditions (Figure 5). The resulting anatomical labeling and the statistical detail are summarized in Table 4. Figure 6 presents the constructed correlation matrices for the sighted and LB groups under the –6 dB SNR and clear speech conditions. The highest connectivity in the sighted group in –6 dB SNR condition was between frontal Fp1 and frontal C3 whereas connectivity in the temporal T7 and parietal Pz, and in the parietal P5 and P3 were highest in the LB group. The highest connectivity in clear speech condition was found Cz-Fp1 and P4-CP6 in the sighted group whereas the highest connectivity was seen in P7-Fp1, Fp1-Fp2, Cz-P8, FC2-P4, CP2-F8, and P3-Fp2 connections in the LB group (Figure 7).

**FIGURE 5:**
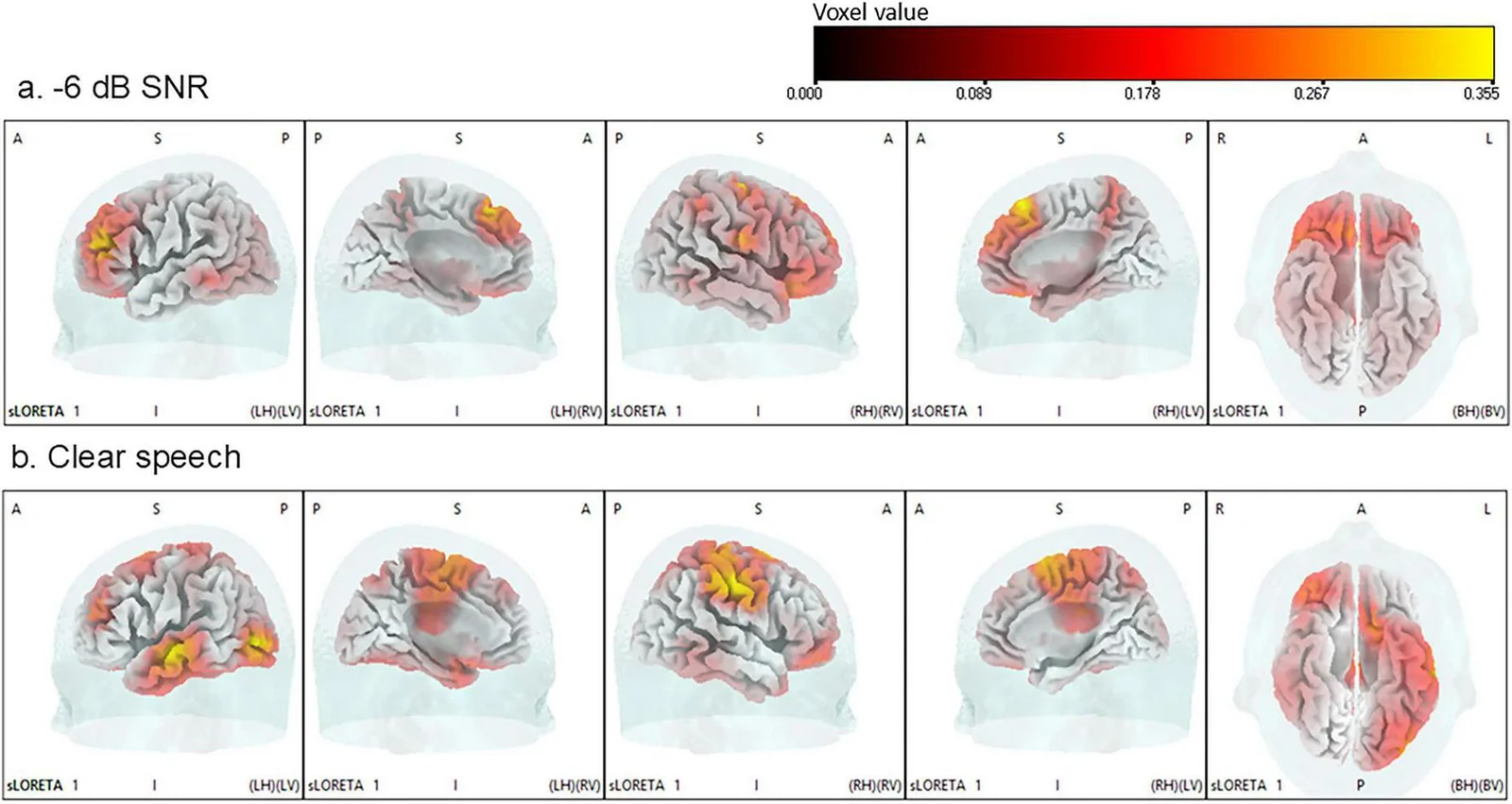
Source localization results using sLORETA under the **(a)** -6 dB SNR and **(b)** clear speech conditions. Yellow areas indicate the highest level of activation, specifically showing greater activation in the sighted group compared to the blind group, while red indicates moderate activation, and gray areas indicate minimal or no activation. Under the -6 dB SNR condition, sighted individuals showed greater activation in frontal regions compared to late blind individuals.

**TABLE 4:** Resulting source map in Sighted > LB in −6 dB SNR and clear speech condition.

Coordinates*			Voxel value	Brodmann area	Lobe	Anatomical labeling (Gyrus)
x	y	z				
−6 dB SNR condition						
−5	32	54	0.355459	6	Frontal	Superior Frontal
−40	−7	60	0.344945	6	Frontal	Precentral
−45	45	16	0.340614	46	Frontal	Middle Frontal
35	−7	46	0.338814	6	Frontal	Middle Frontal
5	36	49	0.338733	8	Frontal	Superior Frontal
−40	40	21	0.33649	10	Frontal	Middle Frontal
−40	35	12	0.334073	46	Frontal	Inferior Frontal
Clear speech condition						
−54	−15	−3	0.169844	21	Temporal	Superior Temporal
−50	−78	4	0.169498	19	Occipital	Middle Occipital
59	−12	42	0.168223	6	Frontal	Precentral
59	−17	42	0.167580	4	Frontal	Precentral
−59	−20	−3	0.167573	21	Temporal	Middle Temporal
59	−13	37	0.167137	4	Frontal	Precentral
64	−18	38	0.166243	3	Parietal	Postcentral
−45	−15	−3	0.165210	22	Temporal	Superior Temporal
-54	−15	−3	0.169844	21	Temporal	Superior Temporal

Coordinates are converted to Talairach space. *p < 0.05, one-tailed. *Coordinates (x, y, z)* indicate the center of activation in Talairach space. *Voxel value* reflects the statistical strength of the source localization at that coordinate. *Brodmann area* and *Anatomical labeling (Gyrus)* are based on standard neuroanatomical atlases.

**FIGURE 6:**
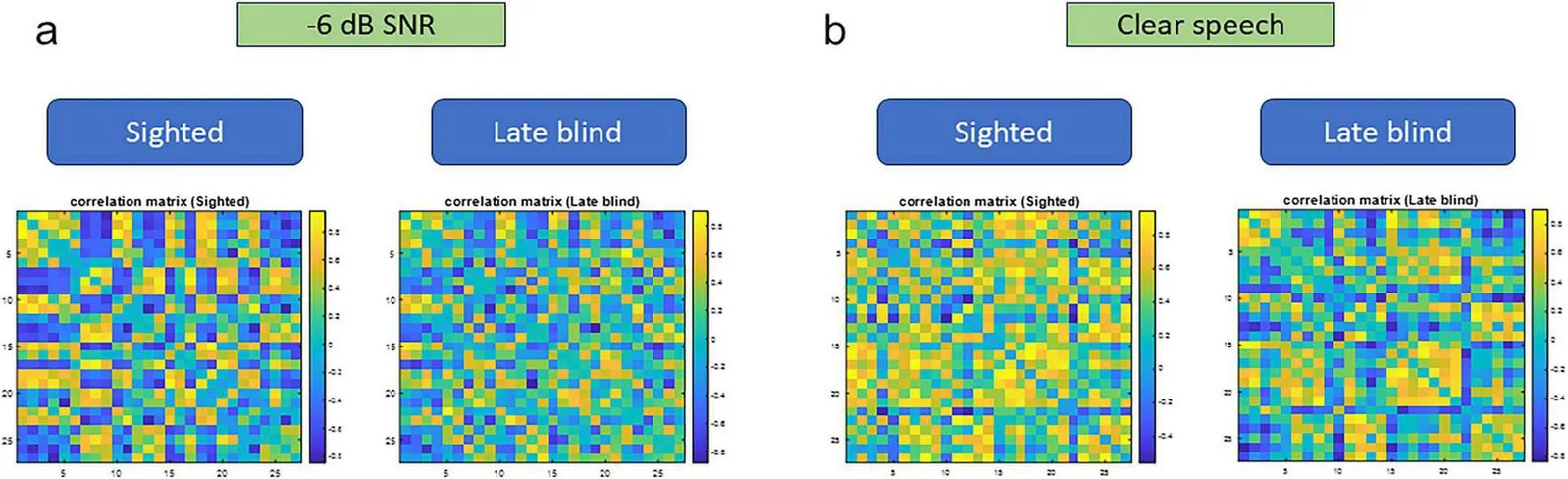
EEG-based correlation matrices in **(a)** -6 dB SNR and **(b)** clear speech conditions. Correlation matrices at the conditions -6 dB SNR (A) and clear speech. The matrices were constructed using 29 electrodes covering the whole brain.

**FIGURE 7:**
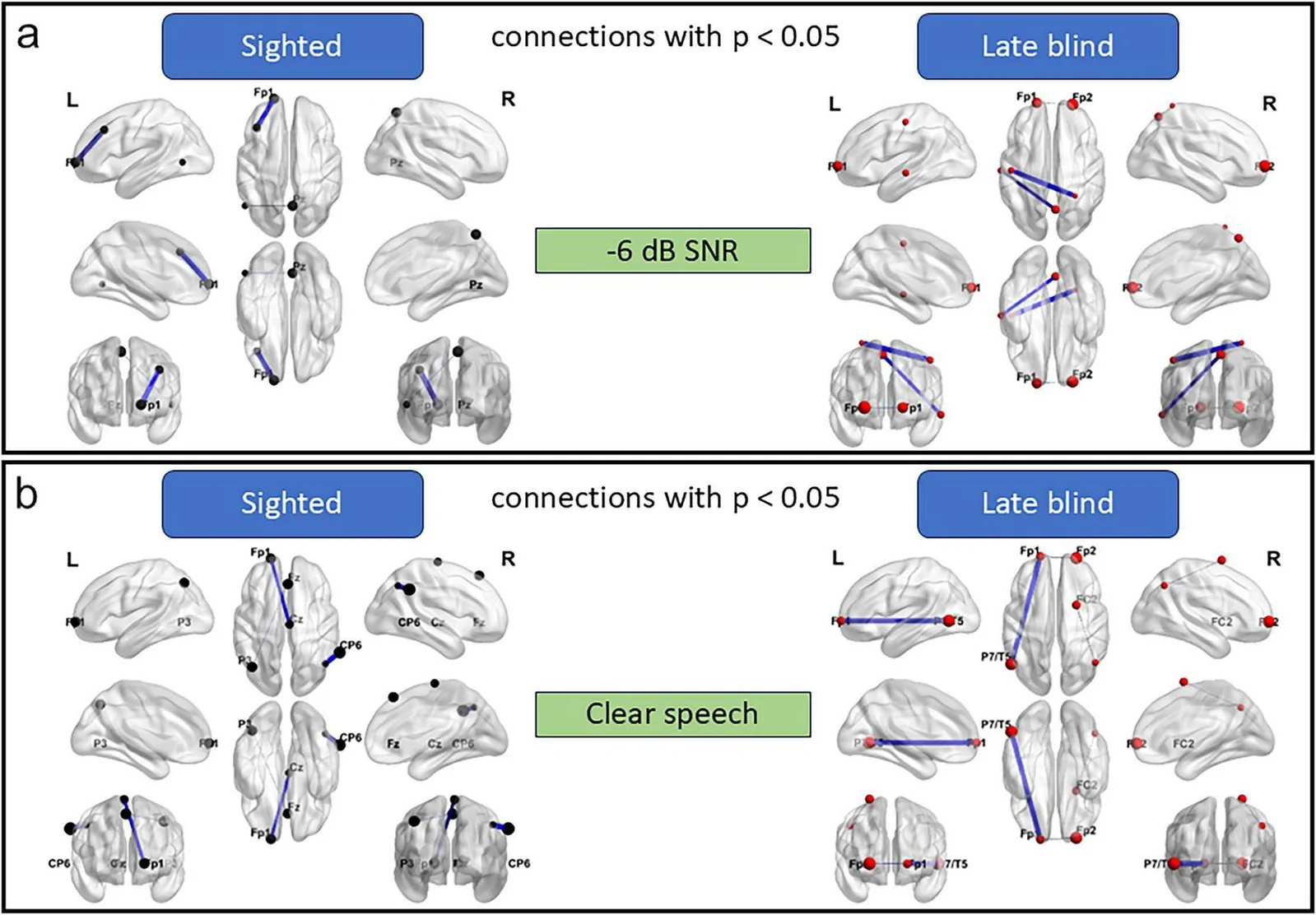
Functional brain connectivity analysis. Connectivity results showing the highest connections (*p* < 0.05) in the sighted and the LB groups in −6 dB SNR condition **(a)** and in clear speech condition **(b)**. Sighted individuals showed relatively frontal connectivity, and late blind individuals showed more widespread connections involving frontal, parietal, and temporal areas. Connections were thresholded at the sparsity of 0.05. Fp1: Frontopolar 1, Fp2: Frontopolar 2, Fz: Frontal midline, F3: Frontal 3 (left frontal), F4: Frontal 4 (right frontal), F7: Frontal 7 (left anterior temporal), F8: Frontal 8 (right anterior temporal), FC1: Fronto-central 1 (left), FC2: Fronto-central 2 (right), Cz: Central midline, P1: Parietal 1 (left parietal), Pz: Parietal midline, CP6: Centro-parietal 6 (right), T5: Temporal 5 (posterior temporal left, also known as P7), P7: Parietal 7 (left lateral parietal).

#### Association between duration of visual loss and auditory behavioral or ERP measures in LB group

No significant correlations were found between the duration of visual deprivation and performance on behavioral auditory tasks, including the Span test, SRD, TMD, sentence recognition in noise, and the oddball task. However, a strong positive correlation was observed between blindness duration and P3b amplitude in the clear condition (r = 0.771, *p* = 0.003). This p-value reflects the Bonferroni correction for multiple comparisons (0.05/4), with an adjusted significance threshold of *p* < 0.0125.

## Discussion

### Summary of key findings

This study investigated how additional sensorineural hearing loss affects auditory-cognitive processing during higher-level speech tasks in blind and sighted individuals, by analyzing behavioral performance and ERP components. Participants with late-onset blindness outperformed sighted individuals in both temporal resolution and working memory tasks. In the oddball tasks, blind participants generally showed higher accuracy across all conditions compared to sighted individuals, although these differences did not reach statistical significance. ERP (N2 and P3b) analysis revealed a significant difference in N2 amplitude at −6 dB SNR, but the effect was in the opposite direction than expected. Source analysis revealed that under adverse listening conditions, the sighted group showed greater dependency on the frontal regions compared to the LB group suggesting compensatory engagement of attentional and working memory resources to support auditory processing. In terms of network connectivity, the sighted group demonstrated strong frontal connectivity under challenging conditions, whereas the LB group showed stronger parietal-temporal connectivity. This finding implies that the LB group can perform the oddball task with less cognitive effort and may still have certain advantages in higher-level speech processing.

### Auditory enhancement and neuronal compensation in blind individuals: evidence from previous studies

Previous studies have demonstrated that blind individuals often exhibit enhanced auditory abilities, supported by both behavioral and electrophysiological evidence. They have demonstrated that EB individuals exhibit enhanced auditory abilities, including superior pitch discrimination (Gougoux et al., 2004), sound localization, and temporal resolution(Röder et al., 1999), along with atypical posterior N2 and mismatch negativity (MMN) responses (Kujala et al., 1995). These findings suggest robust cross-modal plasticity, whereby visual areas are functionally repurposed for auditory processing, and demonstrate that structural and functional cortical reorganization can enhance sensory processing capabilities (Voss and Zatorre, 2012).

As such, the vast majority of prior studies have focused on individuals with congenital blindness. In contrast, some perspectives suggest that in studies involving LB individuals who lost their sight in adulthood, cross-modal recruitment of the visual cortex is more limited compared to the congenitally blind, and no distinct sensory enhancement is observed (Voss et al., 2008). However, other studies simultaneously highlight that LB individuals exhibit unique and differentiated patterns of adaptation based on their past visual experience (Heller, 1989). It is argued that because they possess an extensive visual framework accumulated prior to adulthood, they demonstrate clear advantages in high-order cognitive tasks – such as map memory, shape matching, and spatial computations – by leveraging visual imagery and categorization. Furthermore, according to fMRI and ERP studies, late-blind individuals also show recruitment of the occipital cortex for processing auditory and non-visual information when performing auditory, linguistic, or spatial cognitive tasks (Burton, 2003).

Several hypotheses have been proposed to explain why blind individuals exhibit enhanced cortical responses and behavioral performance. A leading explanation is cross-modal plasticity, where the visual cortex is repurposed to process auditory information, enhancing spatial and temporal resolution. At the onset of visual impairment, the occipital visual cortex is not dormant; it remains actively engaged in functional processing (Choi et al., 2024). Functional compensation also plays a role, as greater reliance on auditory input leads to more refined auditory skills through repeated use. This reflects use-dependent plasticity, where frequent stimulation strengthens specific neural circuits. Additionally, reallocation of attention and cognitive resources toward auditory stimuli contributes to more efficient sensory processing in the absence of visual input.

The studies mentioned above were conducted on blind individuals with “normal hearing”. In contrast, the present study differs in that it focuses on cases of dual sensory loss, where visual impairment co-occurs with sensorineural hearing loss. The neuroplastic changes that occur under such conditions remain largely unexplored. It is still unclear to what extent compensatory mechanisms are preserved or diminished when auditory input begins to decline, or whether only specific auditory functions are retained. Such investigations are rare, and there is a critical need to examine how dual sensory deprivation affects compensatory neural plasticity, especially in aging populations.

### Effects of age-induced hearing impairment on auditory compensation in blind individuals

*A prior* study utilized vocoder-based simulations to mimic spectrally and temporally degraded auditory conditions in blind individuals with normal hearing, thereby modeling the effects of hearing impairment in a controlled experimental setting (Choi et al., 2024). These experiments, conducted primarily on young blind adults, examined auditory-cognitive processing and neural responses (e.g., N2, P3b) under artificially induced hearing loss scenarios. The findings showed how blind individuals sustain enhanced auditory performance and cortical activation even in auditory degraded environments.

This study holds significance in that it directly investigates older blind individuals with confirmed sensorineural hearing loss, evaluating auditory-cognitive performance and neurophysiological responses under actual sensory decline. By going beyond simulated hearing loss models, it offers one of the earliest empirical insights into the effects of dual sensory loss on auditory-cognitive integration and compensatory neural plasticity in aging adults.

A distinctive finding in our study is the superior performance of the LB group stands out in tasks related to temporal modulation and working memory. These behavioral findings build directly upon our previous observations of a selective advantage in temporal modulation detection TMD in LB with normal hearing comparing to the sighted individuals (Shim et al., 2019). Remarkably, the present study demonstrates that this selective advantage in temporal resolution – a capacity known to rely heavily on central auditory cortical mechanisms rather than peripheral spectral tuning – along with superior working memory performance, persists in LB individuals even when compounded by age-related sensorineural hearing loss. One explanation for the selective advantage in temporal resolution in the LB with age-induced sensorineural hearing loss is that neuroplastic changes induced by visual loss preferentially enhance temporal resolution. Since temporal resolution relies more heavily on central auditory mechanism than spectral resolution, even LB individuals – despite limited visual experience – may benefit from cortical plasticity, which helps preserve or enhance temporal processing capacities (Füllgrabe et al., 2015). Moreover, temporal processing often involves higher-order mechanisms such as sequencing, working memory, attention, and prediction, which may benefit from prior visual experience. The selective advantage of LB individuals in temporal, but not spectral, processing may reflect the distinct cognitive demands of the operation of higher-level neural processes. Cattaneo and Vecchi (2011) reported that LB individuals outperform congenitally blind individuals in certain cognitive tasks due to their previous visual input (Cattaneo and Vecchi, 2011). Postma et al. found that while both EB and LB individuals outperformed sighted participants in speeded object-matching tasks, only the LB group demonstrated superior verbal descriptions of spatial layouts (Postma et al., 2007). This suggests that prior visual experience, when combined with sufficient haptic experience, enhances explicit spatial memory and categorical coding.

Another perspective on our findings provides an additional implication that the advantages in spectral resolution and speech recognition seen in young blind individuals (Shim et al., 2019) may be weakened by the emergence of sensorineural hearing impairment. Furthermore, although the sample size of the EB group was relatively small, our behavioral evaluations revealed important insights: specifically, the advantages previously confirmed in EB individuals in previous literature – such as those in SRD, TMD, speech-perception, and odd-ball tasks – are attenuated or lost in this cohort. Also, unlike the findings of previous vocoder-based simulations where blind individuals consistently maintained clear behavioral advantages over the sighted group, the present study observed that the relative superiority of the blind group in the oddball task was attenuated or no longer statistically significant in the presence of actual sensorineural hearing loss.

These findings raise the possibility that dual sensory deprivation may negatively impact cortical reorganization. Although a single ERP paradigm may not fully capture global neural plasticity, the observed behavioral and neural responses offer useful insights into auditory processing in blindness. Our results suggest that auditory processing in blind individuals does not necessarily reflect a fixed or long-term structural reorganization of the brain. While early visual deprivation is typically thought to induce fundamental cross-modal reorganization of the visual cortex (Barulli and Stern, 2013), the reduction in neuroplastic indicators following sensorineural hearing loss implies that such plasticity may be dynamic and dependent on continuous sensory input. Alternatively, the auditory advantages observed in visually deprived individuals may be more accurately interpreted as “use-dependent plasticity” – which is driven by practice and prone to regression without ongoing input – rather than permanent cross-modal reallocation (Makin et al., 2013; Li, 2016).

This potentially flexible nature of sensory-driven plasticity is aligned with short-term deprivation studies. For instance, Pitskel et al. (2007) observed that increased visual cortex responsiveness to auditory stimuli after visual deprivation rapidly diminished upon light re-exposure, pointing to the reversible nature of cross-modal activation. Similarly, Merabet et al. (2008) reported that visual cortex activation during tactile tasks waned once visual input was restored, supporting the idea that sustained sensory exposure may be necessary to maintain these neural adaptations.7

### Divergent brain connectivity patterns between LB and sighted individuals with hearing loss

Under adverse listening conditions (–6 dB SNR), sighted controls exhibited greater recruitment of left frontal regions and stronger fronto-central connectivity compared to the LB group. In cognitive neuroscience literature, it is well established that sighted individuals naturally recruit frontal cognitive-control regions to support speech perception under noise as a standard compensatory strategy involving top-down attentional allocation and executive effort. The lack of a normal-hearing control baseline makes it difficult to definitively determine the source of this group difference. Specifically, we cannot confirm whether it stems from increased compensatory effort in the sighted group or enhanced efficiency in the LB group. However, prior neuroimaging studies on normal-hearing individuals under degraded listening conditions (e.g., noise-vocoded speech or low SNR) consistently show pronounced frontal recruitment – particularly in the DLPFC and IFG – reflecting heightened listening effort and cognitive load (Erb et al., 2013; Du et al., 2016; Alain et al., 2018). This literature-based context supports the view that the strong frontal activation observed in our sighted group aligns with typical cognitive strain under sensory degradation. Thus, the empirical pattern remains clear: the sighted group requires substantially greater frontal cognitive recruitment to handle degraded auditory input, whereas LB individuals engaged a more distributed parietal-temporal network without overtaxing frontal executive control. Combined with their superior working memory and temporal resolution, this distinct neural pattern suggests that LB individuals can process challenging auditory tasks with significantly less cognitive strain despite dual sensory decline.

### Clinical implications

This study explored auditory compensation in blind individuals with sensorineural hearing loss. By focusing on this unique population, the research extends beyond the individual effects of blindness or hearing loss alone, providing clinical insight into the challenges of dual sensory impairment in an aging society. In doing so, it highlights the need for tailored, multisensory rehabilitation strategies for older adults facing compounded sensory decline.

These considerations highlight the need to discuss the implementation of early hearing screening programs for anticipated sensorineural hearing loss in visually impaired individuals. In blind people, early intervention for sensorineural hearing loss with hearing assistance such as hearing aids or cochlear implants may help prevent the regression of neuronal processing in the enhanced visual cortex, emphasizing the importance of managing sensory deficits proactively. Alternatively, visual limitations can hinder the ability to perform speech reading or non-verbal communication through observation of lip movement, facial expression, or other body gestures. Blind individuals face challenges with non-verbal communication and handling hearing devices; therefore, hearing assistive technologies should minimize visual reliance by incorporating tactile and auditory feedback, supported by customized training and assistance programs.

The outstanding working memory and temporal resolution observed in the blind individuals with hearing loss in this study suggests a potentially positive role in their rehabilitation. Clinical interventions should move beyond single-sensory training and incorporate cognitive strategy-based rehabilitation programs, including attention training, working memory enhancement, and predictive processing exercises. In addition, our findings raise further questions about that warrant investigation. It is important to determine whether the decreased compensatory auditory enhancement and adaptive neural changes in blind with hearing impairment is primarily influenced by the regression of the neuroplasticity or by aging brain itself. To address this question, future studies should compare elderly blind individuals with normal hearing thresholds to young blind individuals with hearing impairment. Thus, a comparison between older blind individuals with preserved auditory function and younger blind individuals with hearing impairment would allow for a clearer delineation of whether the regression of compensatory neural adaptation is primarily attributable to sensory deprivation or to intrinsic age-related neurodegenerative processes.

### Methodological considerations and limitations

A primary limitation is the absence of normal-hearing control groups, which precludes establishing a definitive causal link between hearing loss and reduced cross-modal plasticity. Built upon established literature showing superior auditory and ERP profiles in normal-hearing blind adults (Shim et al., 2019; Bae et al., 2022; Choi et al., 2024), our findings should be interpreted as reflecting the distinct behavioral and neural phenotypes of dual sensory impairment, rather than longitudinal degradation driven by hearing loss.

This study focused on older adults with dual sensory loss – specifically, individuals with both visual and auditory impairments – a population that is inherently challenging to recruit due to its rarity and associated vulnerabilities. Particularly noteworthy is the inclusion of a subgroup with early onset visual loss. While numerically limited, this group experiences heightened social isolation, underscoring the importance of their representation. However, due to the limited sample size, statistical analyses for this early onset group were minimized, and the primary source analyses focused exclusively on comparing the late onset group with sighted controls. Consequently, these results necessitate a cautious interpretation, with due consideration of potential confounding variables and contextual factors, emphasizing effect size trends rather than statistical significance.

Additionally, a potential limitation of this study lies in the onset sequence of sensory loss among the participants. The majority of individuals with LB in our cohort experienced vision loss prior to the onset of hearing loss. Consequently, our findings may not be directly generalizable to individuals who experienced hearing loss before vision loss, as the cross-modal reorganization and behavioral outcomes could differ. Furthermore, due to practical recruitment challenges, the age distribution and sex ratio across groups were not well balanced, which may have introduced potential confounding effects on cognitive and electrophysiological measures. Future studies should not only recruit larger cohorts to systematically examine diverse onset sequences, but also incorporate more rigorous designs that strictly match age and sex distributions or statistically control for these demographic factors as covariates.

## Conclusion

In this study, we investigated whether the auditory superiority resulting from visual deprivation in blind individuals with normal hearing is preserved despite the onset of sensorineural hearing loss. While LB individuals with hearing loss demonstrated preserved advantages in specific temporal and working memory tasks, the broader perceptual benefits – particularly in spectral resolution and speech perception in noise – along with robust ERP enhancements were attenuated. In light of prior literature documenting robust auditory enhancements in normal-hearing blind populations, our findings suggest that concurrent age-related hearing loss may limit the full functional expression of compensatory auditory networks. This study highlights the neural and behavioral implications of dual sensory loss in older adults, emphasizing the need to consider them a high-risk group and to provide cognitive-based auditory rehabilitation strategies.

## Data Availability

The original contributions presented in the study are included in the article/Supplementary material, further inquiries can be directed to the corresponding author.

## Supplementary material

Supplementary Figure 1 The figure shows the spectra of spectral-ripple stimuli in the same frequency band. The first panel shows standard and inverted ripples with 0.5 ripples/octave (with logarithmic amplitude), and the second panel shows standard and inverted ripples with 1 ripple/octave. The blue solid lines and red dashed lines represent standard and inverted ripples, respectively.
